# Supplementary material for: Combined Effects of Lycopene and Metformin on Decreasing Oxidative Stress by Triggering Endogenous Antioxidant Defenses in Diet-Induced Obese Mice
Source: Molecules. 2022 Dec 3;27(23):8503. doi: 10.3390/molecules27238503 (PMC9737677; doi:10.3390/molecules27238503)
Supplement: Supplementary file 1 [file molecules-27-08503-s001.zip › molecules-2031572-supplementary.pdf]

**Supplementary Table**

**Table S1.** Compositions of control diet (C) and high-fat diet (HFD).

| **Ingredients** | **Control diet (C) (g/100 g)** | **High-fat diet (HFD) (g/100 g)** |
|---|---|---|
| Starch | 42.75 | 14.95 |
| Casein | 20 | 20 |
| Dextrin | 13.2 | 10 |
| Sucrose | 10 | 10 |
| Soy oil | 4 | 4 |
| Lard | -- | 31 |
| Cellulose | 5 | 5 |
| Mix of minerals [(1)] | 3.5 | 3.5 |
| Mix of vitamins [(2)] | 1 | 1 |
| L-cystine | 0.3 | 0.30 |
| Choline bitartrate | 0.25 | 0.25 |
| Total | 100 | 100 |
| **Energy (kcal/100 g)** | **385** | **540** |

[(1)] Mix of minerals: calcium, phosphorus, potassium, magnesium, iron, manganese, selenium, zinc, chromium, nickel, lithium, sulfur, copper, iodine, molybdenum, silicon, chloride, fluoride, boron, and vanadium.

[(2)] Mix of vitamins: nicotinic acid, calcium pantothenate, pyridoxine, thiamine, riboflavin, folic acid, vitamin K, D-biotin, vitamin B12, vitamin A, vitamin D3, vitamin E, and choline.
